# Crystal Structure and Magnetism of Noncentrosymmetric Eu_2_Pd_2_Sn

**DOI:** 10.1021/acs.inorgchem.1c00678

**Published:** 2021-05-24

**Authors:** Mauro Giovannini, Ivan Čurlík, Riccardo Freccero, Pavlo Solokha, Marian Reiffers, Julian Sereni

**Affiliations:** † Department of Chemistry, University of Genova, Via Dodecaneso 31, 16146 Genova, Italy; ‡ Faculty of Humanities and Natural Sciences, University of Prešov, 17 Novembra 1, 080 01 Prešov, Slovakia; § Institute of Experimental Physics, Slovak Academy of Science, Watsonova 47, 040 01 Košice, Slovakia; ∥ Department of Physics, CAB-CNEA, CONICET, IB-UNCuyo, 8400 S. C. de Bariloche, Argentina

## Abstract

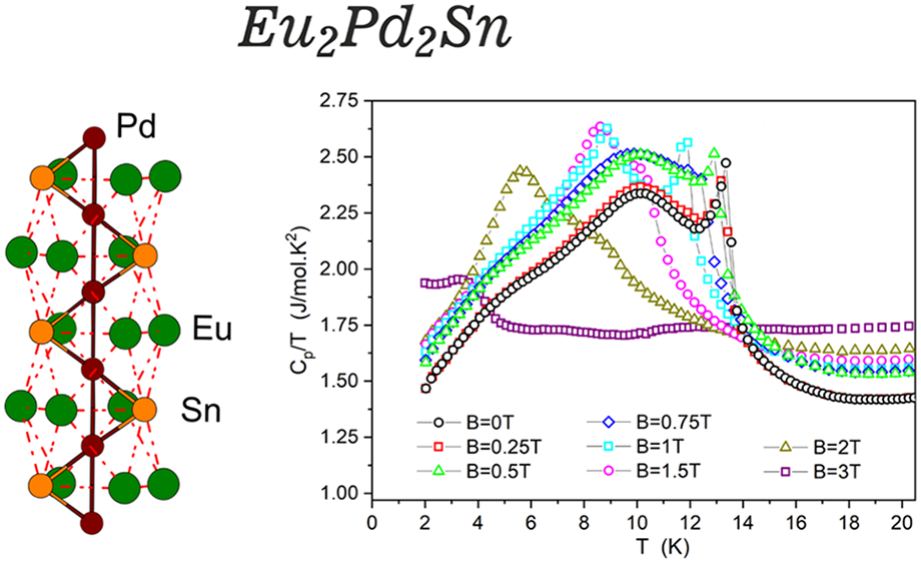

The new intermetallic compound Eu_2_Pd_2_Sn has been investigated. A single crystal was selected from the alloy and was analyzed by single-crystal X-ray diffraction, revealing that this compound possesses the noncentrosymmetric Ca_2_Pd_2_Ge structure type being, so far, the only rare-earth-based representative. Bonding analysis, performed on the basis of DOS and (I)COHP, reveals the presence of strong covalent Sn–Pd bonds in addition to linear and equidistant Pd–Pd chains. The incomplete ionization of Eu leads to its participation in weaker covalent interactions. The magnetic effective moment, extracted from the magnetic susceptibility *χ(T)* is *μ*_eff_ = 7.87 μ_B_, close to the free ion Eu^2+^ value (*μ*_eff_ = 7.94 μ_B_). The maximum of *χ(T)* at *T*_N_ ∼ 13 K indicates an antiferromagnetic behavior below this temperature. A coincident sharp anomaly in the specific heat *C*_P_(*T*) emerges from a broad anomaly centered at around 10 K. From the reduced jump in the heat capacity at *T*_N_ a scenario of a transition to an incommensurate antiferromagnetic phase below *T*_N_ followed by a commensurate configuration below 10 K is suggested.

## Abstract 2

A new intermetallic compound, Eu_2_Pd_2_Sn, is so far the only rare-earth representative of the Ca_2_Pd_2_Ge structure type. Synthesis and characterization of the structure, together with magnetic properties, are discussed.

## Introduction

Intermetallic compounds based on rare earth elements, such as Ce, Eu, and Yb with different configurations of their f-electrons, show a wealth of fascinating properties.^1−3^ These systems are potentially interesting mainly due to the anomalous properties of these three rare earths, which are not always in the trivalent (3+) state like the majority of other rare earths. An interesting case comprises compounds based on Eu^2+^ (and, analogously, Gd^3+^) with a pure spin configuration of *J* = *S* = 7/2 and *L* = 0, which show the absence of crystal electric field (CEF) effects. Surprisingly, instead of a negligible magnetic anisotropy, Eu intermetallics frequently exhibit a complex and strongly anisotropic magnetism.^4,5^ In some cases, namely for EuNiGe_3_ and EuPdSn, despite the substantial neutron absorption of Eu, a large-area flat-plate geometry was used to thoroughly investigate the magnetic structure of Eu-based intermetallics. In both compounds, a complex incommensurate antiferromagnetic scenario with a thermal evolution of the magnetic structure was described in detail.^6,7^

Several other compounds belonging to the Eu–Pd–Sn system have been studied, namely Eu_3_Pd_2_Sn_2_, EuPdSn_2_, EuPd_2_Sn_4_, and EuPd_2_Sn_2_, all showing a stable Eu^2+^ magnetic state and complex magnetic structures.^8−10^

From the perspective of compositions and crystal structures, we dedicated some systematic work to the study of the R_2_Pd_2_X (R = Ce, Yb; X = In, Sn) compounds with the tetragonal Mo_2_FeB_2_ structure type.^11,12^ The Mo_2_FeB_2_ structure is the most representative (more than 200 compounds) of the systems with the general composition (AE/R)_2_T_2_X (AE = alkaline earth metals or R = lanthanide block, T = late transition metals, and X = Mg, Zn, Cd, Al, Ga, In, Sn, Pb). Due to the remarkable electronic flexibility of this group, many of the compounds crystallizing in the Mo_2_FeB_2_ type exhibit outstanding physical properties.^13−15^ Notably, no representative of this structure type is known with divalent rare earth metals like Eu and only a few containing Yb^16,17^ or the chemically similar alkaline earth metals. This is probably due to the bigger sizes of these metals, which stabilize other structure types. For instance, there are only two 2:2:1 Eu compounds, namely, Eu_2_Pd_2_In and Eu_2_Pt_2_In, crystallizing in the monoclinic HT-Pr_2_Co_2_Al type with distinctly different polyanionic networks.^18^ The same occurs replacing Eu by Ca or Sr, whereas Ca_2_Pd_2_Cd forms with a W_2_B_2_Co-type structure.^19^

In the present work, we report on the existence of a third Eu compound of the 2:2:1 family, namely Eu_2_Pd_2_Sn, which crystallizes in the noncentrosymmetric orthorhombic Ca_2_Pd_2_Ge-type structure. Noteworthy, Eu_2_Pd_2_Sn is the only rare-earth representative of this structure type. Moreover, magnetic systems lacking inversion symmetry are a materials class of special interest, because the antisymmetric Dzyaloshinskii–Moriya (DM) interactions are allowed and they may stabilize magnetic structures with a unique chirality and nontrivial topology.^20^ Therefore, we have investigated the structural, magnetic, electrical, and thermal properties and electronic structure of this compound.

## Experimental Section

### Sample Preparation

The metals used as starting materials were palladium (foil, 99.95 mass% purity, Chimet, Arezzo, Italy), tin (bar, 99.999 mass% purity, New Met Koch, Waltham Abbey, U.K.), europium (pieces, 99.99% mass, Smart-Elements GmbH, Vienna, Austria). The sample, with a total weight of 1 g, was prepared by weighting in a glovebox under Ar atmosphere the proper amount of elements by using an analytical balance.

In order to avoid the loss of europium during melting due to its high vapour pressure, the stoichiometric amount of the starting elements was enclosed in a small tantalum crucible sealed by arc welding in inert atmosphere inside the glovebox. The sample was subsequently melted in an induction furnace under a stream of pure argon. To ensure homogeneity, the crucible was continuously shaken during melting. The sample was then annealed in a resistance furnace for 1 week at 900 °C and finally quenched in cold water.

The sample was characterized by scanning electron microscopy (SEM) supplied by Carl Zeiss SMT Ltd., Cambridge, England, and by electron probe microanalysis (EPMA) based on energy dispersive X-ray spectroscopy. For quantitative analysis an acceleration voltage of 20 kV was applied for 100 s, and a cobalt standard was used for calibration. The X-ray intensities were corrected for ZAF effects.

### Single-Crystal X-ray Diffraction Analysis

An Eu_2_Pd_2_Sn single crystal was selected from the alloy with the aid of a light optical microscope (Leica DM4000 M, Leica Microsystems Wetzlar GmbH, Wetzlar, Germany) operating in the dark-field mode. A full-sphere data set was obtained in a routine fashion under ambient conditions on a three-circle Bruker Kappa APEXII CCD area-detector diffractometer equipped with graphite monochromatized Mo Kα (λ = 0.71073 Å) radiation operating in ω-scan mode. Intensities were collected over the reciprocal space up to ∼30° in θ, with an exposure time of 30 s per frame. Semiempirical absorption corrections based on a multipolar spherical harmonic expansion of equivalent intensities were employed for all data using the SADABS software.^21^ Details about the crystal structure solution and refinement are reported in the Results and Discussion section.

### Electronic Structure Calculations

The Eu_2_Pd_2_Sn electronic structure was studied by means of the TB-LMTO-ASA 4.7c program,^22,23^ employing the Barth–Hedin^24^ exchange and correlation potential within the local density approximation (LDA). The space filling was reached without the addition of interstitial empty spheres. The calculations were performed with the following atomic spheres radii: *r*(Eu) = 2.133 Å, *r*(Sn) = 1.616 Å, *r*(Pd) = 1.431 Å. The basis set included Eu-6*s*/6*p*/5*d*, Sn-5*s*/5*p*/5*d*/4*f*, and Pd-5*s*/5*p*/4*d*/4*f* orbitals, with Eu-6*p*, Sn-5*d*/4*f*, and Pd-4*f* functions being downfolded. The Eu 4*f* wave functions were treated as core states occupied by 7 electrons which results in formal Eu(II) valence, consistent with physical properties measurements. The Brillouin zone was sampled through a set of 1160 irreducible *k*-points out of 4096.

Chemical bonding investigations were conducted on the basis of the obtained density of states (DOS), crystal orbital Hamilton populations (COHP), and their integrated values (ICOHP) up to the *E*_F_; the corresponding curves were plotted with the wxDragon software.^25^

### Magnetic and Thermal Measurements

Magnetic susceptibility, magnetization, heat capacity, and electrical resistivity were measured by the cryogen-free physical property measurements system DYNACOOL commercial device (Quantum Design) in the temperature range of 2–300 K and in an applied field up to 9 T. For heat capacity measurements the two-τ model of the relaxation method was used. Electrical resistivity was carried out using a standard four-probe technique.

## Results and Discussion

### Crystal Structure of Eu_2_Pd_2_Sn

Structure refinement parameters together with selected crystallographic data for the studied Eu_2_Pd_2_Sn single crystal are listed in Table 1. Further details on the crystal structure investigations may be obtained from the Cambridge Structural Database on quoting the depository number also indicated in Table 1.

**Table 1:** Crystallographic Data and Experimental Details of the Structure Determination for the Eu_2_Pd_2_Sn Single Crystal

empirical formula	Eu_2_Pd_2_Sn
CSD depository number	1975626
structure type	Ca_2_Pd_2_Ge
space group	*Fdd*2 (no. 43)
Pearson symbol, *Z*	*oF*40, 8
*hkl* range	±14; ±23; ±8
unit cell dimensions:	
*a*, Å	10.4741(4)
*b*, Å	16.0712(6)
*c*, Å	5.8718(2)
*V*, Å^3^	988.41(6)
calcd density (*D*_calc,_ g cm^–3^)	8.54
abs. coefficient (μ, mm^–1^)	36.93
extinction coefficient	0.00049(2)
Flack parameter	0.033(15)
total no. reflections	7940
GOF	1.1
independent reflections	846 (*R*_int_ = 0.0152)
reflections with *I* > 2σ(*I*)	799 (*R*_sigma_ = 0.0253)
data/parameters	26/846
*R* indices (*I* > 2σ(*I*)); *R*_1_/*wR*_2_	0.0109/0.0123
*R* indices (all data)	0.0197/0.0200
Δρ_fin_ (max/min), e nm^–3^ (× 10^3^)	0.66/–0.60

Cell indexation was straightforward for Eu_2_Pd_2_Sn, giving an orthorhombic *F*-centered cell (*h + k* = 2*n*; *k* + *l* = 2*n*, and *h* + *l* = 2*n* reflections were observed). The analysis of systematic extinctions suggests the only possible space group *Fdd*2 (no. 43). A chemically reasonable structural model was obtained in a few iteration cycles by applying the charge-flipping algorithm implemented in JANA2006.^26^ In this model the rare earth and Pd atoms are situated in different 16*b* sites of general symmetry, whereas the remaining Sn atoms occupy the 8*a* site.

Further structure refinements were carried out by full-matrix least-squares methods on |*F*^2^| using the SHELX programs^27^ as implemented in WinGX.^28^ The anisotropically refined Eu_2_Pd_2_Sn model showed acceptable residuals and flat difference Fourier maps. The absolute structure for Eu_2_Pd_2_Sn was ensured through the refinement of the Flack parameter. Obtained atomic positions and equivalent isotropic displacement parameters are listed in Table 2.

**Table 2:** Atomic Positions and Equivalent Isotropic Displacement Parameters for Eu_2_Pd_2_Sn

atom	site	*x/a*	*y/b*	*z/c*	*U*_iso_ (Å^2^)
Eu	16*b*	0.16851(2)	0.04827(2)	0.08315(8)	0.0119(1)
Sn	8*a*	1/2	0	0.08963(8)	0.0095(1)
Pd	16*b*	0.66749(3)	0.12492(2)	0.07505(6)	0.0133(1)

**Figure 1:**
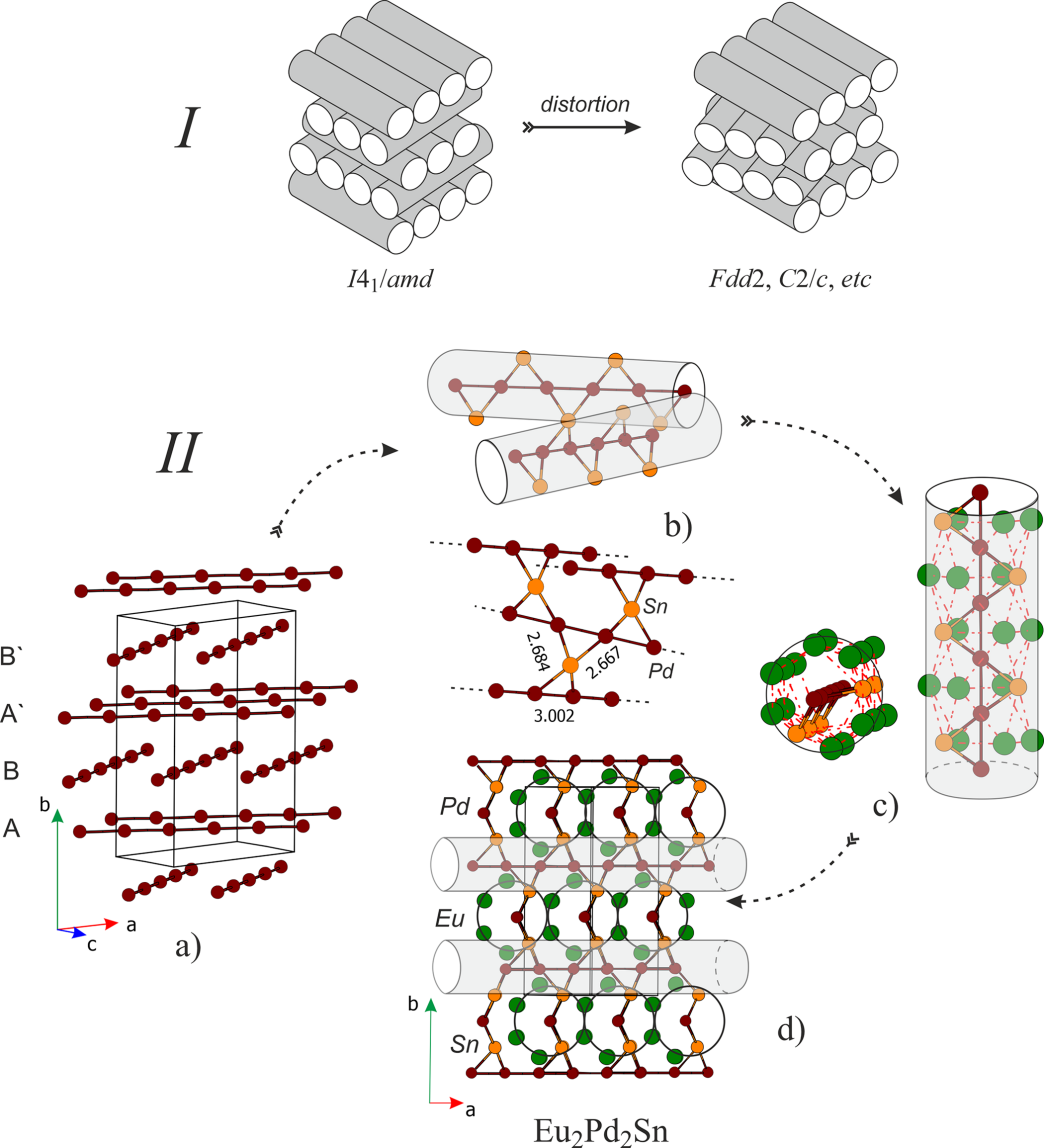
(I) Schematic representation of body-centered tetragonal layer packing of regular cylinders and its distortion variant; (II) crystal structure of Eu_2_Pd_2_Sn: (a) packing and relative orientation of the Pd linear chains, (b) spatial distribution of SnPd_4_ distorted tetrahedra joining adjacent Pd chains, (c) unique cylinder-like structural fragment of Eu_2_Pd_2_Sn composition, and (d) Eu_2_Pd_2_Sn unit cell projection viewed along the [101] direction highlighting the complex Pd–Sn network and distribution of Eu atoms.

As already mentioned in the Introduction, there are several 2:2:1 stoichiometry structural types that count with hundreds of representatives (for example, Mo_2_FeB_2_, W_2_CoB_2_, etc.). Two dozen compounds of the general formula (AE/R)_2_T_2_X crystallize either in *mS*40-Ca_2_Ir_2_Si (SG: *C*2/*c*) or in *oF*40-Ca_2_Pd_2_Ge structure type (SG: *Fdd*2). They contain similar structural fragments and the same local arrangement for analogous species.^29^ Moreover, for Sr_2_Pd_2_Al the possibility that both polymorphs with the above-mentioned structure exist was reported.^30^ Despite a detailed structural description of these compounds, an alternative presentation, highlighting the crystal chemical similarities, might be conducted to exploit the idea of rod packings extensively applied by ÒKeeffe.^31^ The clarity of the crystal chemistry representation when the “rods” of atoms are considered as a structural moiety was also shown for several intermetallic compounds.^32,33^ Here, we would like to use this idea to depict the structural features of the title compound. It is well-known that one of the closest packings of undistorted cylinders is the tetragonal base centered one of *I*4_1_/*amd* symmetry. With a distortion of the rod fragment, naturally, the total number of symmetry elements reduces and the obtained derivative structures may not necessarily follow group–subgroup relations, as it was emphasized by Doverbratt et al.^29^ for Ca_2_Pd_2_Ge and Ca_2_Pt_2_Ge (see scheme I in Figure 1).

For Eu_2_Pd_2_Sn the most remarkable structural feature is the presence of almost linear Pd–Pd chains where palladium atoms are distanced at 3.002 Å (similarly as in Sr_2_Pd_2_Al).^30^ These chains could be considered as the axis of Eu_2_Pd_2_Sn rods shown in Figure 5 1a–c. The rod itself as well as the crystal structure do not possess inversion symmetry elements; this feature makes Eu_2_Pd_2_Sn attractive for magnetic interactions studies. The dihedral angle between two chains from adjacent layers is ca. 65.2°. The Pd chains are fused between themselves through Sn bridging atoms in the form of strongly distorted SnPd_4_ tetrahedra (their distribution finally defines the symmetry of the compound, see Figure 1b). The interatomic Sn–Pd distances of ∼2.67 Å are close to the sum of the constituent’s covalent radii, indicating strong interactions, as discussed more in detail in the next paragraph. Far from the center, each rod fragment is enveloped by the biggest Eu atoms as shown in Figures 1c and 1d. The Eu–Pd contacts vary between 3.14 and 3.27 Å; the Eu–Sn ones are distanced at 3.48–3.65 Å, whereas Eu–Eu atoms are distanced by 3.73, 3.86, and 3.89 Å between first, second, and third neighbors, respectively.

### Chemical Bonding

**Figure 2:**
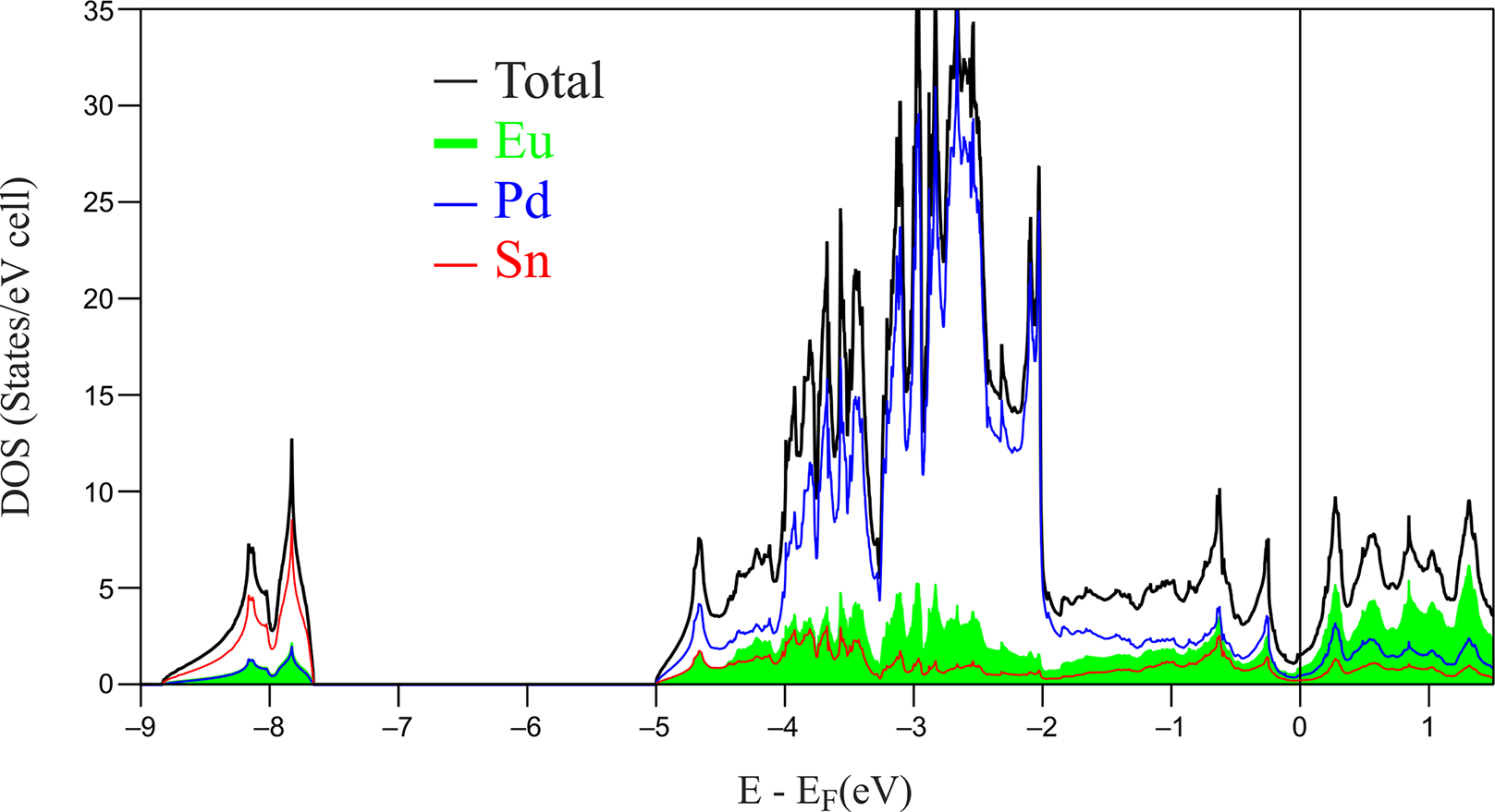
Calculated total and projected density of states (pDOS) for Eu_2_Pd_2_Sn.

This is the third intermetallic compound, together with Ca_2_Pd_2_Ge^29^ and Sr_2_Pd_2_Al,^30^ crystallizing in the *oF*40-Ca_2_Pd_2_Ge structure (SG: *Fdd*2). Since DOS/COHP-based chemical bonding studies were already performed for both of the previously reported compounds, a comparative analysis is appropriate, in particular with the isovalent Ca_2_Pd_2_Ge. All of them show very similar DOS (see Figure 2 for Eu_2_Pd_2_Sn), with the *E*_F_ located in a deep pseudogap for both Eu_2_Pd_2_Sn and Ca_2_Pd_2_Ge compounds. In the case of Sr_2_Pd_2_Al, the reduced number of valence electrons (28 vs 27 v.e. per formula unit) push the *E*_F_ close to a local maximum.^30^

The lowest-lying states in Eu_2_Pd_2_Sn are mainly the Sn-4*s* (for all the orbital *p*DOS see the Supporting Information). The large peak between −4 and −2 eV is primarily dominated by Pd-4*d* states considerably mixing with the Sn-4*p* and Eu states, suggesting polar covalent interactions. The presence of a strong Eu contribution to the occupied states is clear evidence of its incomplete ionization and participation in chemical bonding, which is a quite common feature for ternary *RE*-tetrelides.^34−37^ The COHP curves (see the Supporting Information) and ICOHP values (see Table 3 and the Supporting Information) show only a few differences that can be highlighted and discussed between Eu_2_Pd_2_Sn and Ca_2_Pd_2_Ge.

**Table 3:** Selected Distances and Average −ICOHP Values for Eu_2_Pd_2_Sn and Ca_2_Pd_2_Ge^29^

Eu_2_Pd_2_Sn			Ca_2_Pd_2_Ge		
atom pair	distances (Å)	–ICOHP (eV/bond)	atom pair	distances (Å)	–ICOHP (eV/bond)
Sn–Pd	2.668(1) and 2.684(1)	1.91	Ge–Pd	2.493(1) and 2.523(1)	2.23
Pd–Pd	3.002(1)	0.85	Pd–Pd	2.8721(1)	0.99
Eu–Sn	3.481(1) to 3.645(1)	0.55	Ca–Ge	3.285(2) to 3.541(1)	0.50
Eu–Pd	3.140(1) to 3.271(1)	0.63	Ca–Pd	3.022(2) to 3.164(1)	0.64

In both cases, the strongest interactions are those between Pd and the tetrel elements (Ge and Sn). For the title compound this value is smaller (−1.91 eV) than in Ca_2_Pd_2_Ge (−2.23 eV), probably due to the longer Pd–Sn distances compared to the Pd–Ge ones. The same is also true for the Pd–Pd bonding, where a distance increase of about 0.13 Å leads to a reduced ICOHP, lowering from −0.99 to −0.85 eV. Almost the same ICOHP value (−0.84 eV) was obtained also in Sr_2_Pd_2_Al where the Pd–Pd distance is 3.031 Å, confirming that Pd atoms are still bonded. Finally, focusing on the interactions of the most electropositive elements (Eu and Ca) with their neighboring species, only tiny differences can be pointed out. In particular, although Eu and Sn are about 0.15 Å further than Ca and Ge, they show almost the same ICOHP values. Interestingly, both Eu–Sn and Eu–Pd COHP curves are practically optimized at *E*_F_, without any occupied antibonding state.

Ca_2_Pd_2_Ge was described as a Zintl-like phase, according to the ionic (Ca^2+^)_2_(Pd^0^)_2_Ge^4–^ formula, predicting semimetallic properties based on the performed DFT calculations.^29^ Despite the similar bonding scenario, the description of Eu_2_Pd_2_Sn as a semimetallic Zintl-like compound seems not to be appropriate. Although electroneutrality is similarly respected by the formula (Eu^2+^)_2_(Pd^0^)_2_Sn^4–^, it is too far from the revealed bonding scenario. For a typical ionic and Zintl phase, where a more effective charge separation occurs, the real situation is much closer to the formal one (i.e., Na^+^Cl^–^; K^+^[(2*b*)As^–^] – 2*b* = “two bonded”). It is not the case for the title compound. In fact, the strongest interactions are the polar covalent Sn–Pd ones, clearly evidencing how approximated the formal assumption of (0*b*)Sn^4–^ species is. Finally, the DOS has low but nonzero states at *E*_F_, and the electrical resistivity measurements (see Figure 5b) indicate a metallic behavior.

### Magnetic Properties

**Figure 3:**
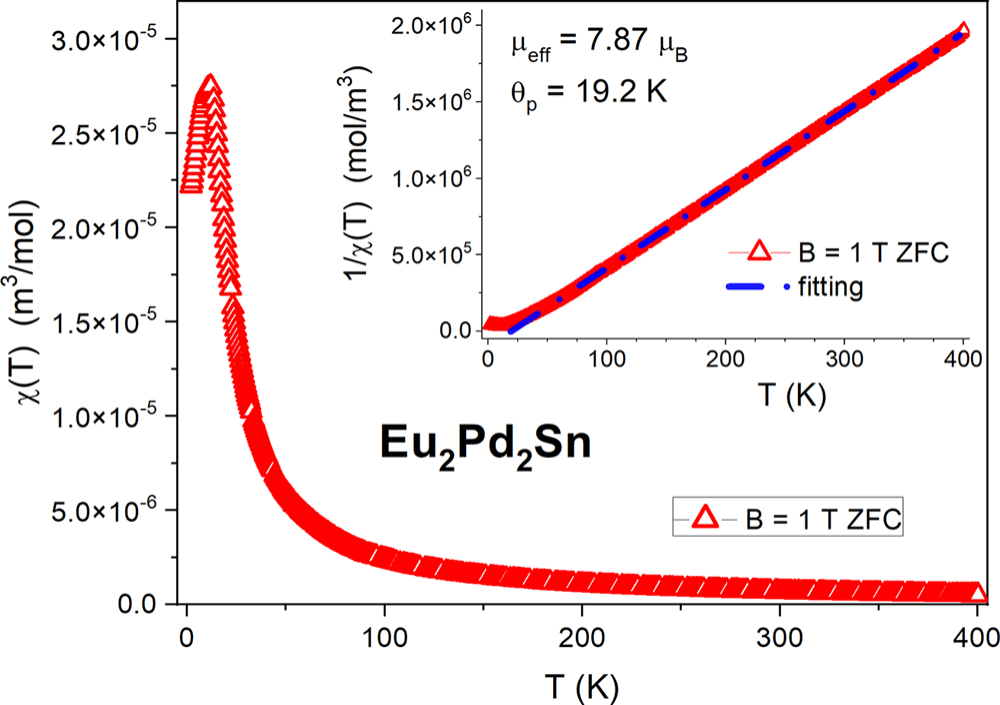
Temperature dependence of the magnetic susceptibility χ(*T*) of Eu_2_Pd_2_Sn in an applied magnetic field of *B* = 1 T, measured in zero-field cooling (ZFC) mode. The inset displays the inverse magnetic susceptibility 1/χ (the blue dash-dotted line represents the Curie–Weiss law fitting).

As displayed in the inset of Figure 3, the inverse magnetic susceptibility 1/χ(*T*) of Eu_2_Pd_2_Sn in the paramagnetic region follows the Curie–Weiss law. The least-squares fitting above 60 K gave an effective moment of μ_eff_ = 7.87 μ_B_ and a paramagnetic Curie–Weiss temperature θ_p_ of 19.2 K. The magnitude of μ_eff_ is close to the free ion Eu^2+^ value (μ_eff_ = 7.94 μ_B_), whereas the positive value of θ_p_ reflects ferromagnetic interactions. Nevertheless, the sharp maximum at 13.3 K (see Figure 3) of the magnetic susceptibility is symptomatic of antiferromagnetic ordering, although a more complex magnetic structure cannot be excluded while also taking into account the positive value of θ_p_ data.

**Figure 4:**
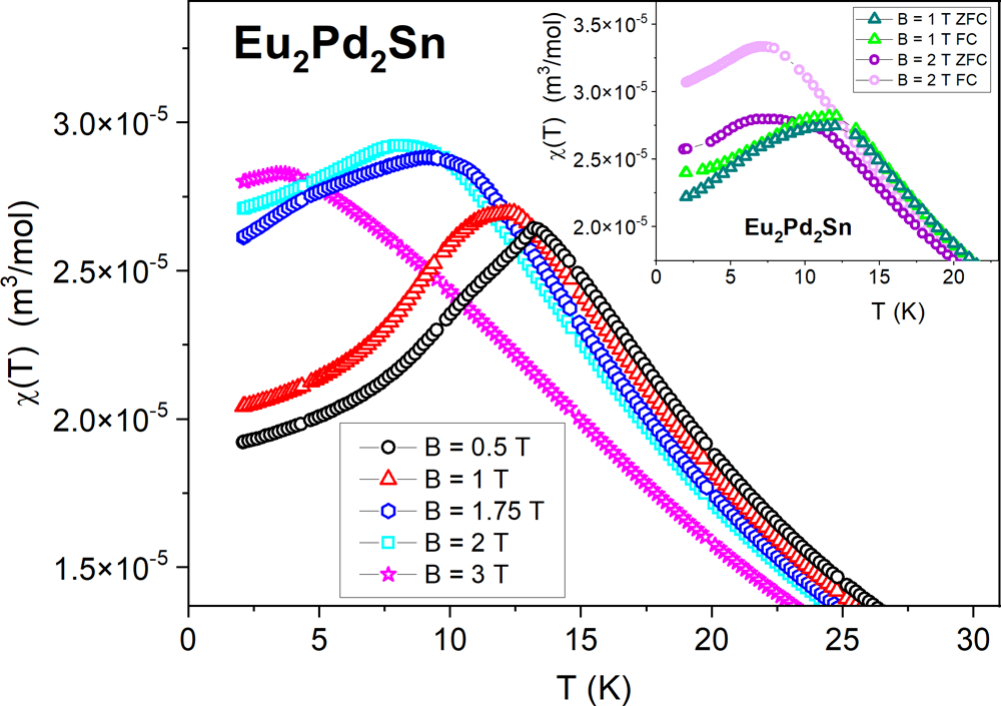
Low-temperature χ(*T*) dependences of Eu_2_Pd_2_Sn for different magnetic fields. In the inset the χ(*T*) plots of ZFC/FC regimes measured for *B* = 1 and 2 T are compared.

Figure 4 presents the χ(*T*) data in the range of 2–25 K for different magnetic fields. For all the curves, by increasing *B* the maxima shift to lower temperatures.

For the lowest applied field of *B* = 0.5 T, a maximum at 13.3 K can be observed, followed by an emerging shoulder at 10 K. This effect reinforces in strengthened magnetic fields, producing a broad maximum around the same temperature for *B* = 1 T and a splitting of both maxima at 1.75 T. Then, for *B* = 3 T, the lower-temperature shoulder practically vanishes. At the same field, χ(*T*) continuously increases by decreasing temperature and then shows a broad hump at around 3.5 K. Therefore, the magnetic phase boundary of Eu_2_Pd_2_Sn cannot be described as purely antiferromagnetic (AFM) because another component is involved in the formation of the maximum of the magnetic susceptibility.

**Figure 5:**
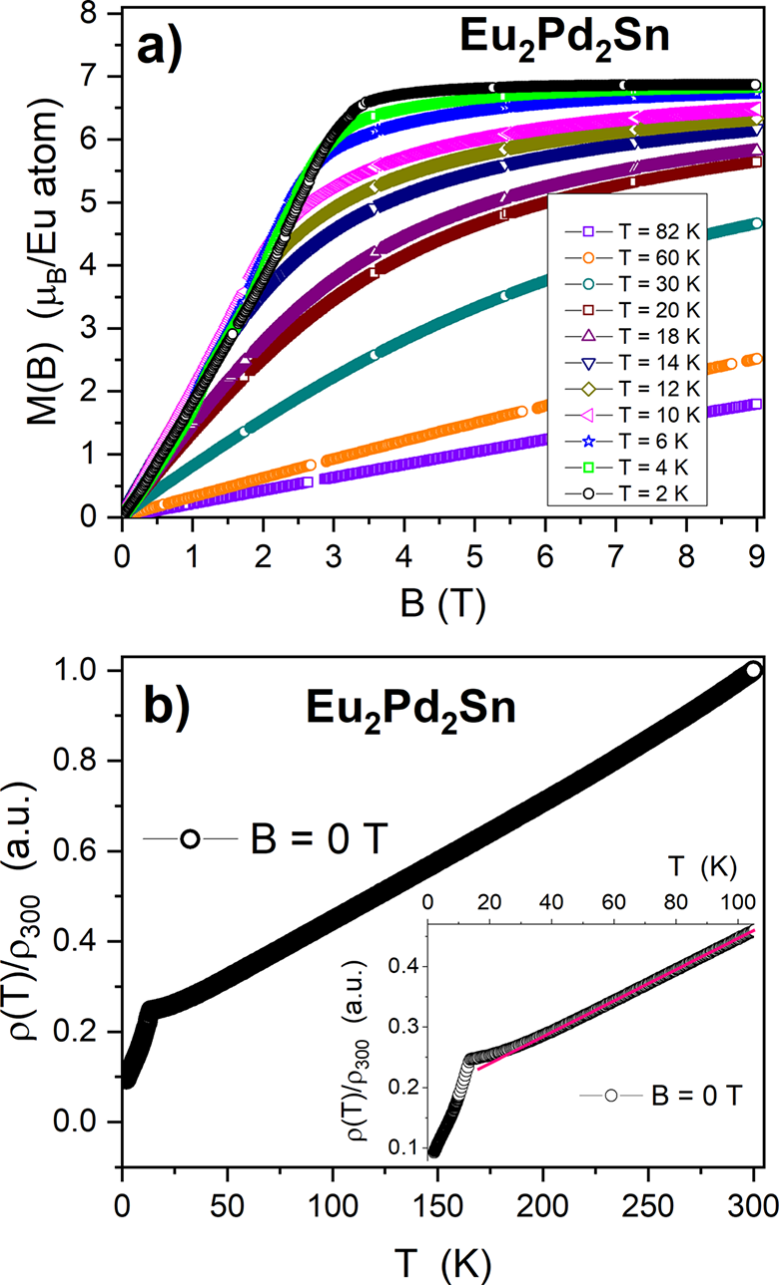
(a) Isothermal magnetization *M*(*B*) of Eu_2_Pd_2_Sn at some selected temperatures from 2 to 82 K. (b) Zero-field electrical resistivity between room temperature and 2 K normalized at room temperature. Inset: detail of the ρ(*T*) curvature above the magnetic transition.

The isothermal magnetization *M(B)* curves at temperatures ranging from 2 to 82 K are shown in Figure 5a. Whereas the curves at 82 and 60 K show paramagnetic behavior, those below 30 K exhibit an increasing curvature which transforms into a significant shoulder below 12 K. The saturation value *M*_sat_ ∼ 6.85 μ_B_/Eu atom (close to the theoretical saturation value of 7 μ_B_/Eu atom for Eu^2+^) is reached at *T* > 4 K for *B* > 6 T. Notably, a slight positive curvature is observed in the *M(B)* isotherm at 2 K for magnetic field values between 1 and 2 T, followed by the rapid saturation in the magnetization for *B* > 3.5 T. This feature is also observed in other Eu^2+^ intermetallics (like EuPtSi and EuPtGe^38^) described as noncentrosymmetric frustrated systems dominated by DM interactions.

The strong increase of *M*(*B*) observed in Figure 5a and the maximum of *M*(*T*) at *T*_N_ look conflicting in terms of a FM or AFM description of the magnetically ordered phase. This scenario can be expected in a FM system presenting domain walls pinning. However, the lack of coercive effects in *M*(*B*) loops (not shown) at low temperature (i.e., 2 K) excludes such possibility. Noncollinear or modulated magnetic order may be considered for this anisotropic compound from which different types of behavior may occur in different crystallographic directions. Similar behaviors of complex and strongly anisotropic magnetism were found in other known Eu intermetallics.^4−9^ Interestingly, there is only a weak difference detected between ZFC and FC measurements below *T*_N_ up to *B* = 1 T, while at *B* = 2 T a clear separation of those curves occurs (see inset of Figure 4). This suggests the formation of field-induced FM regions within the system in an external field of 2 T as an intrinsic property of its magnetic structure.

**Figure 6:**
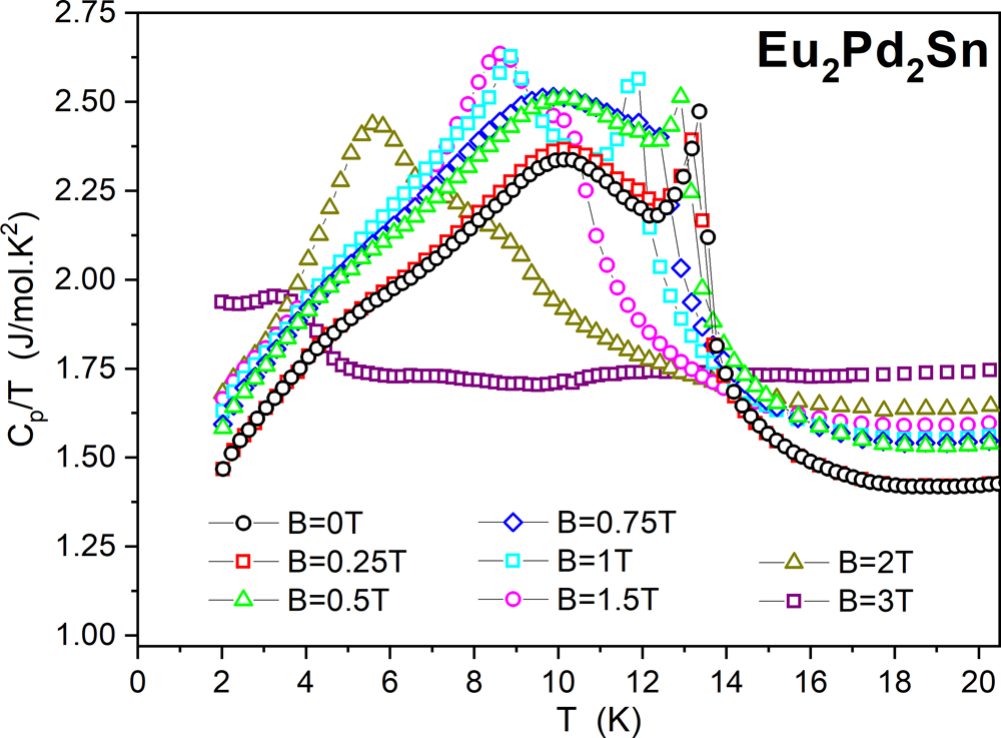
Temperature dependence of heat capacity divided by temperature, *C*_P_/*T*, of Eu_2_Pd_2_Sn for different magnetic fields.

The normalized temperature dependence of the electrical resistivity of Eu_2_Pd_2_Sn is presented in Figure 5b. The residual resistivity ratio RRR ∼ 10 indicates a rather acceptable quality of the polycrystalline sample. The ρ(*T*) decreases monotonously with decreasing temperature as occurs in ordinary metals. In the inset, the low-temperature region of the electrical resistivity does not exhibit the expected cusp for an antiferromagnetic transition. Instead, a tail develops up to about 60 K, and above this temperature, the resistivity becomes linear. Below the transition, a slight change of slope is observed around 10 K, in correspondence with the broad anomaly observed in *C*_P_/*T* (see Figure 6). Since below that temperature ρ(*T*) follows a nearly linear *T* dependence, the change of slope may be related to a change of electronic scattering regime, from one dominated by critical fluctuations (around *T*_N_) to a coherent one at lower temperature.

Specific heat measurements as a function of temperature, depicted as *C*_P_/*T*, are reported in Figure 6 for different applied magnetic fields up to *B* = 3 T. In zero magnetic field, a sharp peak around 13.4 K is related to the antiferromagnetic transition shown in χ(*T*). This peak seems to emerge from a broad anomaly centered at around 10 K. Notably, similar narrow peaks are found in the heat capacity measurements of other noncentrosymmetric Eu compounds, either in isotropic compounds like in EuPtX (X = Si, Ge)^38^ or in anisotropic structures such as EuPtSi_3_^39^ and EuNiGe_3_.^40^ In the first case it was attributed to strong fluctuations, whereas in the second it was associated with an incommensurate antiferromagnetic intermediate phase that is followed by a commensurate phase transition at ∼10 K.^40^ Also in the case of Eu_2_Pd_2_Sn, it can be attributed to a rearrangement of the Eu magnetic structure which correlates with the tiny anomalies found in the magnetic susceptibility and resistivity measurements at the same temperature. In fact, the specific-heat jump at *T*_N_ is estimated as Δ*C*_P_ ∼ 14 J/mol K at zero field. This value is clearly smaller than the value prediction (20 J/mol K) in the mean-field approximation for a magnetic structure with equal moment (EM) for the 8-fold degenerated *J* = 7/2 ground state of Eu^2+^. Nevertheless, our estimation of the value of Δ*C*_P_ is in very good agreement with the prediction (13.4 J/mol K) in the mean-field approximation for an incommensurate amplitude modulated (AM) magnetic structure which results in a decrease of 2/3 compared to the EM case.^39,41^

Under applied magnetic fields, up to *B* = 1 T, the temperature and the height of the jump Δ*C*_P_ at 13.4 K are only slightly reduced, whereas for higher fields, it transforms in a shoulder of the broad anomaly at *T* ∼ 10 K. This last one remains practically unaffected by the field for *B* < 1 T; however, for *B* = 1 and 1.5 T, it shifts to lower temperature without changing its height in *C*_P_(*T*). For *B* = 3 T, it practically smears out around 3.8 K.

Finally, the broad anomaly at around 4 K is common in Eu^2+^ and Gd^3+^ systems and is related to the large degeneracy of the *J* = 7/2 local moment.^41^

**Figure 7:**
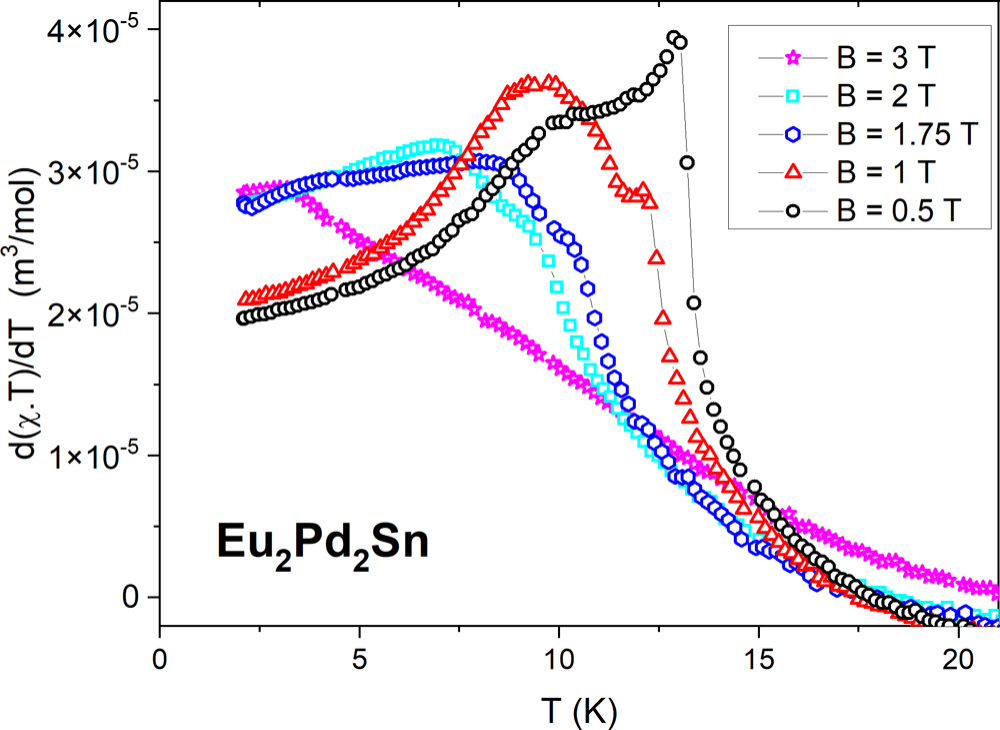
Temperature dependence of the d(χ*T*)/d*T* derivative of Eu_2_Pd_2_Sn for selected applied magnetic fields.

The analysis of the complex behavior of the magnetically ordered phase depicted by the specific heat in Figure 6 can be complemented by the magnetic susceptibility data expressed as the d(χ*T*)/d*T* derivative shown in Figure 7. Notice that, in Figure 6, the specific heat is presented as *C*_P_*/T*, whereas the d(χ*T*)/d*T* representation corresponds to the internal magnetic energy *U*_m_ derivative, i.e., *C*_m_, excluding phonon contribution. The full magnetic character of the transition at 13.4 K is confirmed by this result. Compared with the specific heat results, this transition rapidly weakens in its magnetic intensity in applied magnetic fields. However, the hump at 10 K shows a nonmonotonous evolution with field in both temperature position and intensity. This anomalous behavior requires a more detailed study of this magnetic field range.

## Conclusions

The novel intermetallic compound Eu_2_Pd_2_Sn has been synthesized and structurally studied by means of X-ray single-crystal diffraction. Eu_2_Pd_2_Sn is the first rare-earth compound crystallizing with the orthorhombic Ca_2_Pd_2_Ge structure type.

The complex scenario revealed by the study of the bonding analysis evidences how much the Zintl-like (Eu^2+^)_2_(Pd^0^)_2_Sn^4–^ formulation is approximated.

The magnetic behavior of this compound is dominated by a robust magnetic Eu^2+^ lattice. The measurements of magnetization, resistivity, and specific heat seem to suggest a scenario of a transition at *T*_N_ from paramagnetism to an intermediate phase which has an incommensurate amplitude modulated (AM) magnetic structure, followed by a commensurate phase transition at around 10 K. This scenario is supported by the value of the jump of this transition Δ*C*_P_ = 14 J/mol K which is very close to the value predicted in the case of an AM magnetic structure. Moreover, the magnetic origin of the transition at around 10 K is supported by the fact that it was found in all the measurements (magnetization included). Applying a magnetic field, the incommensurate transition seems to gradually disappear starting from values of *B* > 1 T, becoming a satellite of the lower temperature anomaly. This scenario could be demonstrated only by further suitable measurements, in particular by neutron diffraction. Finally, the apparent contradiction of a positive value of the paramagnetic Curie–Weiss temperature may be attributed to exchange anisotropy or to a competition between ferromagnetic and antiferromagnetic interactions.

## Supporting Information Available

Details on interatomic distances and ICOHP for Eu_2_Pd_2_Sn; total and orbital projected DOS for Sn, Pd, and Eu atoms; and COHP curves for Sn–Pd, Sn–Eu, Pd–Pd, and Pd–Eu (PDF)

## Accession Codes

CCDC 1975626 contains the supplementary crystallographic data for this paper. These data can be obtained free of charge via www.ccdc.cam.ac.uk/data_request/cif, or by emailing data_request@ccdc.cam.ac.uk, or by contacting The Cambridge Crystallographic Data Centre, 12 Union Road, Cambridge CB2 1EZ, UK; fax: +44 1223 336033.
