# Supplementary material for: Crystal Structure and Magnetism of Noncentrosymmetric Eu2Pd2Sn
Source: Inorg Chem. 2021 May 24;60(11):8085–92. doi: 10.1021/acs.inorgchem.1c00678 (PMC8277132; doi:10.1021/acs.inorgchem.1c00678)
Supplement: Supplementary file 1 — ic1c00678_si_001.pdf [file ic1c00678_si_001.pdf]

Supporting Information

Crystal Structure and Magnetism of Noncentrosymmetric Eu_2Pd_2Sn

Mauro Giovannini*[,a], Ivan Čurlík[b], Riccardo Freccero[a], Pavlo Solokha[a], Marian Reiffers[b,c], Julian Sereni[d]

[a]Department of Chemistry, University of Genova, Via Dodecaneso 31, Genova, Italy
[b]Faculty of Humanities and Natural Sciences, University of Prešov, 17.novembra 1, Prešov, Slovakia
[c]Institute of Experimental Physics, Slovak Academy of Science, Watsonova 47, Košice, Slovakia
[d]Department of Physics, CAB-CNEA, CONICET, IB-UNCuyo, 8400 S. C. de Bariloche, Argentina
*Email: mauro.giovannini@unige.it

Table S.1. Interatomic distances (< 3.7 Å) and Integrated (up to the E_F) Crystal Orbital Hamilton Population (ICOHP) for Eu_2Pd_2Sn.

Central atom	Adjacent Atoms	*d* (Å)	–ICOHP (*eV/bond*)	Central atom	Adjacent atoms	*d* (Å)	–ICOHP (*eV/bond*)
**Eu**	Pd	3.140	0.73	**Pd**	Sn	2.668	1.89
	Pd	3.228	0.56		Sn	2.684	1.92
	Pd	3.233	0.67		Pd(x2)	3.002	0.85
	Pd	3.254	0.64		Eu	3.140	0.73
	Pd	3.259	0.54		Eu	3.228	0.56
	Pd	3.271	0.61		Eu	3.233	0.67
	Sn	3.481	0.73		Eu	3.254	0.64
	Sn	3.544	0.45		Eu	3.259	0.54
	Sn	3.558	0.55		Eu	3.271	0.61
	Sn	3.645	0.47	**Sn**	Pd(x2)	2.668	1.89
					Pd(x2)	2.684	1.92
					Eu(x2)	3.481	0.73
					Eu(x2)	3.544	0.45
					Eu(x2)	3.558	0.55
					Eu(x2)	3.645	0.47

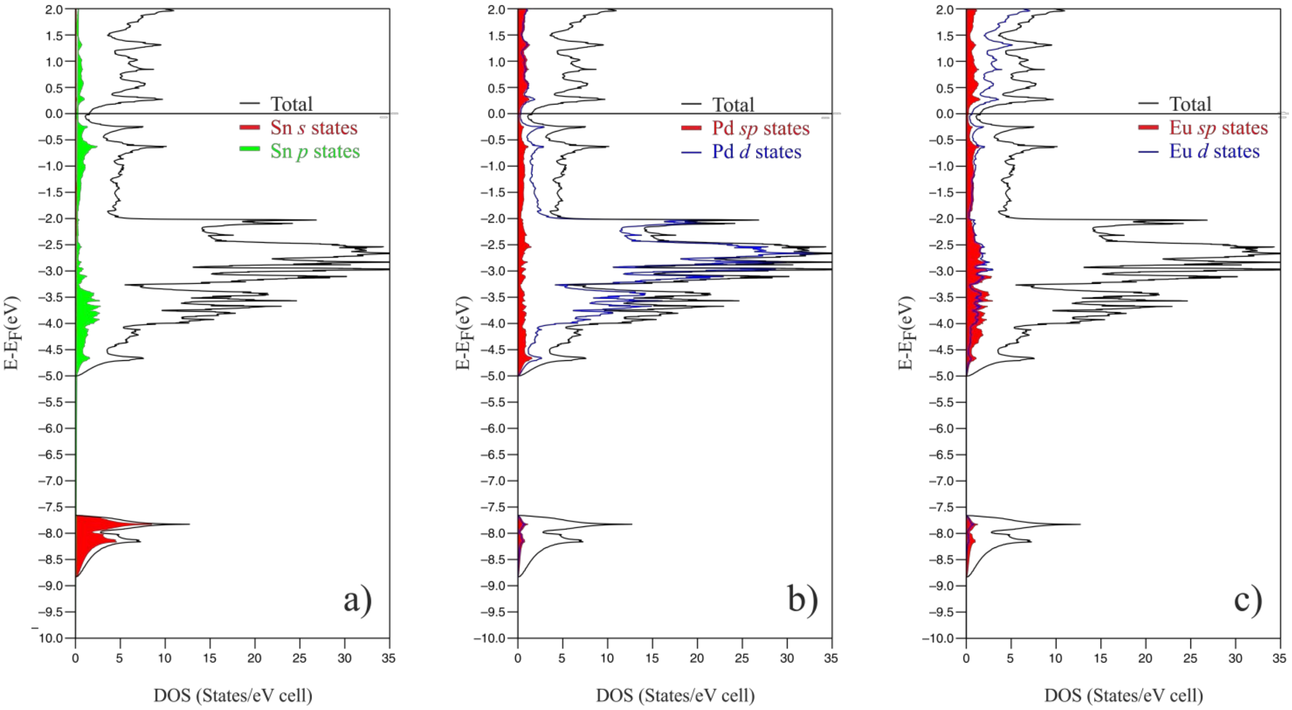

Fig. S.1. Total and orbital projected DOS for Sn (a), Pd (b) and Eu (c) atoms.

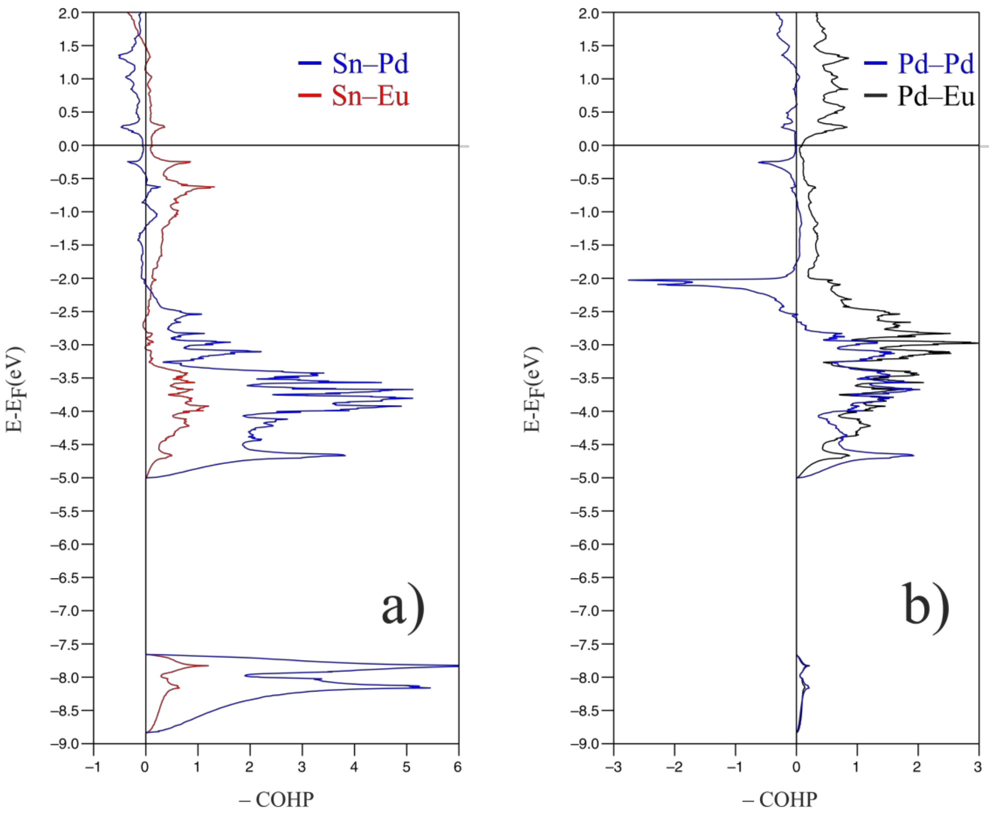

Fig. S.2. Crystal Orbital Hamilton Populations (–COHP curves) for Sn–Pd, Sn–Eu (a) and Pd–Pd, Pd–Eu (b).
